# Mirikizumab is equally effective in ustekinumab-exposed and ustekinumab-naïve patients with UC: a multicenter real-world cohort

**DOI:** 10.1093/crocol/otag065

**Published:** 2026-06-19

**Authors:** Mohmmed Tauseef Sharip, Dharmaraj Durai, Eva Vayreda, Nurulamin M Noor, Anushka Herath, Achini Withanachchi, Robin Dart, Sreedhar Subramanian, Miles Parkes, Mark A Samaan, Tim Raine, Peter M Irving

**Affiliations:** Department of Gastroenterology, Cambridge University Hospital NHS Trust, Cambridge, United Kingdom; Department of Medicine, University of Cambridge, Cambridge, United Kingdom; Gastroenterology Department, Cardiff and Vale University Health Board, Cardiff, United Kingdom; Department of Gastroenterology, Guy’s and St Thomas’ NHS Foundation Trust, London, United Kingdom; Department of Gastroenterology, Cambridge University Hospital NHS Trust, Cambridge, United Kingdom; Department of Medicine, University of Cambridge, Cambridge, United Kingdom; Gastroenterology Department, Cardiff and Vale University Health Board, Cardiff, United Kingdom; Gastroenterology Department, Cardiff and Vale University Health Board, Cardiff, United Kingdom; Department of Gastroenterology, Guy’s and St Thomas’ NHS Foundation Trust, London, United Kingdom; Department of immunobiology, School of Immunology and Microbial Sciences, King’s College London, United Kingdom; Department of Gastroenterology, Cambridge University Hospital NHS Trust, Cambridge, United Kingdom; Department of Gastroenterology, Cambridge University Hospital NHS Trust, Cambridge, United Kingdom; Department of Medicine, University of Cambridge, Cambridge, United Kingdom; Department of Gastroenterology, Guy’s and St Thomas’ NHS Foundation Trust, London, United Kingdom; Department of Gastroenterology, Cambridge University Hospital NHS Trust, Cambridge, United Kingdom; Department of Gastroenterology, Guy’s and St Thomas’ NHS Foundation Trust, London, United Kingdom; Department of immunobiology, School of Immunology and Microbial Sciences, King’s College London, United Kingdom

**Keywords:** mirikizumab, ulcerative colitis, ustekinumab, p19 inhibitors

## Abstract

**Background:**

Mirikizumab, a selective IL-23p19 monoclonal antibody, has demonstrated efficacy in moderate-to-severe ulcerative colitis (UC) although real-world data are limited. In addition, registrational trials excluded patients previously treated with ustekinumab, which targets the IL-12/23 p40 subunit. We aimed to evaluate the real-world effectiveness and safety of mirikizumab in a multicenter UK cohort of patients with UC, including those with prior ustekinumab exposure.

**Methods:**

A retrospective observational study was conducted across 3 tertiary UK centers. Adults with confirmed UC who received mirikizumab and had ≥12 weeks of follow-up were included. Data on demographics, disease activity (SCCAI, UCEIS), biomarkers (CRP, fecal calprotectin), and treatment persistence were analyzed at 3 and 6 months. Outcomes were compared between ustekinumab-exposed and ustekinumab-naïve patients.

**Results:**

Among 100 patients (36 ustekinumab-exposed), treatment persistence at 3 months was 72% with no significant difference between groups. SCCAI scores decreased from 5.8 to 3.3 at 3 months (*P* < .00001), and fecal calprotectin decreased from 1094 µg/g to 586 µg/g (p = 0.006). UCEIS scores (*n* = 36) improved significantly post-induction (mean 4.4 to 2.7, *P* < .000001). Multivariable logistic regression did not reveal any factors associated with treatment persistence, including prior ustekinumab exposure. Adverse events were infrequent and mild.

**Conclusion:**

Mirikizumab is effective and well tolerated in a real-world UC population, including patients with prior ustekinumab exposure. These findings support the use of IL-23p19 inhibition regardless of previous IL-12/23 blockade.

## Graphical abstract

**Figure:**
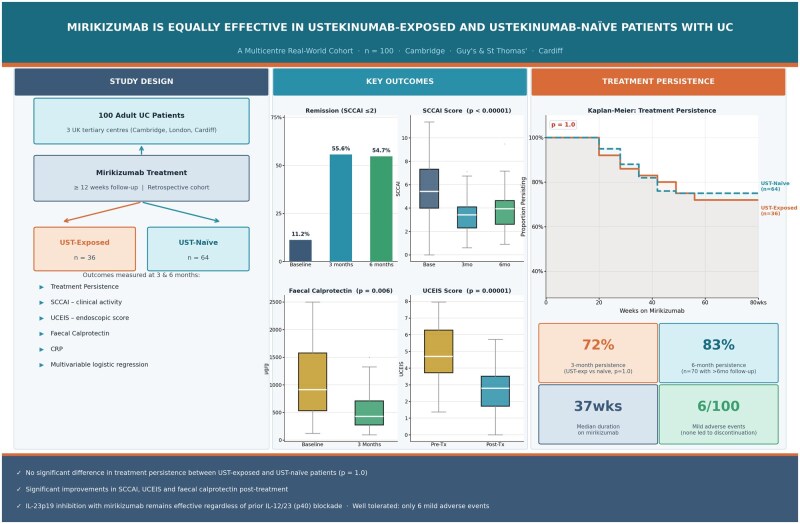

**What is already known?** 

- Mirikizumab is an IL-23p19 monoclonal antibody effective in inducing and maintaining remission in moderate-to-severe ulcerative colitis (UC).
- The pivotal LUCENT trials demonstrated its efficacy in biologic-experienced patients but excluded those previously treated with ustekinumab.
- Current treatment guidelines recommend switching to a therapy with a different mechanism of action after biologic failure.
- There is limited real-world evidence assessing the effectiveness of mirikizumab, particularly in ustekinumab-exposed UC patients.

**What are the new findings?** 

- Mirikizumab demonstrated similar clinical, biochemical, and endoscopic responses in ustekinumab-exposed and ustekinumab-naïve patients with UC.
- Treatment persistence at 3 and 6 months was high (72%) and was unaffected by prior ustekinumab exposure.
- Fecal calprotectin and endoscopic scores significantly improved, confirming mucosal response in addition to symptom relief.
- This is the largest real-world cohort with the longest follow-up evaluating mirikizumab in UC and includes a significant cohort of ustekinumab-exposed patients.

**How can this study help patient care?** 

- These data will help clinicians and patients considering treatment with mirikizumab to contextualize its use in a real-world setting providing evidence of clinical and endoscopic response.
- They also reinforce the appropriateness of mirikizumab in anti-tumour necrosis factor (anti-TNF)-exposed patients and, crucially, in ustekinumab-exposed patients.
- Patients and clinicians should be reassured that the safety profile is similar to that seen in the clinical trial program.

## Introduction

Since the introduction of anti-TNF agents for the treatment of inflammatory bowel disease (IBD) over 20 years ago, several agents with varying mechanisms of action, including both biologics and small molecules, have been introduced. The most recent class of therapy for ulcerative colitis (UC) is the anti-p19 monoclonal antibodies, of which mirikizumab was the first to be licensed. Mirikizumab is a humanized IgG4-variant monoclonal antibody that binds the p19 sub-unit of interleukin (IL)-23, and was shown in phase 2 and 3 trials to induce and maintain remission in patients with moderate to severe UC.^1–3^

Importantly, the phase 3 registration studies (LUCENT 1 and 2) excluded patients with previous ustekinumab exposure, a monoclonal antibody targeting the p40 subunit shared by IL-12 and IL-23, which thus also results in IL-23 inhibition.^4^ Recent guidelines on the management of patients with AT failure in IBD recommend switching to a treatment with a completely different mechanism of action.^5^^,^^6^ However, limited data in Crohn’s disease suggest that IL-23 inhibition may be effective in patients who have previously been exposed to ustekinumab.^7^^,^^8^ Such data are, however, largely lacking in UC; therefore, the appropriateness of using mirikizumab in UC patients with prior ustekinumab exposure remains uncertain.

We, therefore, aimed to understand the effectiveness of mirikizumab in a real-world cohort of patients with UC, including patients who had previously been exposed to ustekinumab.

## Methods

We conducted a retrospective observational study to assess the treatment persistence and real-world effectiveness of mirikizumab across 3 large tertiary referral centers in the UK: Cambridge University Hospital, Guy’s and St Thomas’ Hospitals, and Cardiff and Vale University Hospital. Consecutive patients treated with mirikizumab were identified through electronic medical records. To ensure a robust assessment of treatment induction, only patients who had reached a minimum of 12 weeks of follow-up were included in the final analysis (*n* = 100). No patients were excluded due to early intolerance or treatment discontinuation prior to this landmark. Data were collected retrospectively from prospectively maintained clinical records. Inclusion criteria were age ≥18 years, with a confirmed diagnosis of UC based on standard clinical, endoscopic, and histopathological evaluation, and at least 12 weeks of follow-up data. Patients with a history of previous total or sub-total colectomy were excluded from the analysis. Demographic data, disease activity measures including the Simple Clinical Colitis Activity Index (SCCAI),^9^ the Ulcerative Colitis Endoscopic Index of Severity (UCEIS),^10^ and biomarkers such as C-reactive protein (CRP) and fecal calprotectin were recorded.

Concomitant steroid use at baseline, along with previous exposure to other AT, including ustekinumab, was documented. Follow-up data were obtained at 3 and 6 months. UCEIS scores at any point post-induction (ie, after 12 weeks) were recorded where available.

### Outcomes

The primary outcome was time to treatment discontinuation assessed by Kaplan–Meier survival analysis over a 70-week follow-up period. As some patients could have an extended induction with mirikizumab, treatment persistence at 6 months was recorded to include all patients who received at least one dose of mirikizumab subcutaneously. Non-persistence was defined as either switching to a different biologic or treatment discontinuation due to non-response, adverse effects, or need for colectomy. Other outcomes included clinical response (≥3-point reduction in SCCAI), clinical remission (SCCAI ≤2), steroid-free remission, endoscopic activity (UCEIS), biomarkers, colectomy rates, and adverse events. Patients who remained on mirikizumab were censored at the time of data analysis.

### Statistical analysis

Treatment persistence was assessed using the Kaplan–Meier survival analysis, with time to event defined as time from mirikizumab initiation to discontinuation; patients remaining on treatment at data extraction were censored at their last known follow-up, and between-group differences were compared using the log-rank test. Response rates for clinical and endoscopic disease activity were calculated using SCCAI and UCEIS, respectively. Baseline demographics and laboratory parameters were summarized using means and standard deviations. Paired analyses were performed for clinical, biochemical, and endoscopic data at the respective time points, excluding patients with missing data. Statistical significance was determined using two-tailed *t*-tests, chi-square, Wilcoxon rank test, Mann–Whitney *U* test, and ANOVA, with an alpha of 0.05. Multivariable logistic regression models evaluated whether baseline characteristics, prior biologic exposure, or previous ustekinumab use influenced persistence on mirikizumab. All analyses were conducted using Python in Jupyter Notebook.

## Results

A total of 100 patients met the inclusion criteria and were included in the analysis. Baseline demographics and disease characteristics are detailed in Table 1. Patients were symptomatic with raised biomarkers; active endoscopic inflammation was noted in 73% in whom baseline endoscopy was available. The remaining 27% of patients with a baseline UCEIS < 2 were switched to mirikizumab due to high symptom burden (mean SCCAI 5.0) and biochemical activity (mean fecal calprotectin 1026 μg/g), to maintain stability following a recent failure of another therapeutic class, or were patients in whom the endoscopy may have occurred several weeks prior to mirikizumab initiation after which clinical relapse occurred.

**Table 1:** Patient demographics and baseline disease characteristics.

Characteristic	Total cohort (*n* = 100)	Prior ustekinumab exposure (*n* = 36)	Ustekinumab-naive (*n* = 64)
**Age, years mean (SD)**	—	47.2 (17.4)	44.1 (14.8)
**Disease extent**			
** Pancolitis**	47 (47%)	13 (36.1%)	34 (53.1%)
** Left-sided colitis**	47 (47%)	20 (55.6%)	27 (42.2%)
** Proctitis**	6 (6%)	3 (8.3%)	3 (4.7%)
**Baseline values, mean (SD)**			
** CRP, mg/L**	—	7.0 (10.4)	9.5 (12.5)
** SCCAI**	—	5.3 (3.0)	6.1 (3.0)
** UCEIS**	—	5.0 (1.5)	4.9 (1.6)
** Fecal calprotectin, µg/g**	—	1026 (1264)	1241 (1239)
**Number of prior advanced therapy exposures**			
** 0**	2 (2%)	0 (0%)	2 (3.1%)
** 1**	24 (24%)	1 (2.8%)	23 (35.9%)
** 2**	30 (30%)	9 (25.0%)	21 (32.8%)
** 3**	28 (28%)	12 (33.3%)	16 (25.0%)
** 4**	11 (11%)	9 (25.0%)	2 (3.1%)
** 5**	2 (2%)	2 (5.6%)	0 (0%)
** 6**	3 (3%)	3 (8.3%)	0 (0%)
** Steroid use at baseline**	38 (38%)	11 (30.6%)	27 (42.2%)
**Induction regimen**			
** Standard induction doses**	59 (59%)	23 (63.9%)	36 (56.2%)
**Previous advanced therapies**			
** Infliximab**	46 (46%)	19 (52.8%)	27 (42.2%)
** Adalimumab**	45 (45%)	17 (47.2%)	28 (43.8%)
** Golimumab**	7 (7%)	4 (11.1%)	3 (4.7%)
** Vedolizumab**	65 (65%)	30 (83.3%)	35 (54.7%)
** Upadacitinib**	10 (10%)	1 (2.8%)	9 (14.1%)
** Filgotinib**	5 (5%)	1 (2.8%)	4 (6.2%)
** Tofacitinib**	21 (21%)	12 (33.3%)	9 (14.1%)
** Ozanimod**	5 (5%)	2 (5.6%)	3 (4.7%)

**Table 2:** Clinical disease activity over time.

**Time point**	Total *N*	Clinical remission (SCCAI ≤2)	Remission rate (%)	Clinical improvement (≥3-point drop)	Improvement rate (%)
**Baseline**	89	10	11.2	—	—
**3 months**	82	50	55.5	44	53.7
**6 months**	52	29	54.7	32	61.5

The cohort was almost exclusively bio-experienced, and 36% had previously been exposed to ustekinumab.

### Treatment persistence and landmark analysis

Drug durability was assessed via Kaplan-Meier survival analysis (Figure 4A). Median persistence was not reached within the 70-week follow-up. Landmark persistence rates were 72% (3 months), 68% (6 months), and 64% (12 months).

The median duration of mirikizumab treatment was 37 weeks (SD: 18). While 59 patients received a standard induction regimen, 41 (41%) underwent extended induction protocols. All patients transitioned to a maintenance dose of 200 mg subcutaneously every four weeks. Sixteen patients (16%) required re-induction with a subsequent three-dose course of intravenous mirikizumab; additionally, 23 patients (23%) received a course of corticosteroids during treatment.

In a multivariable logistic regression model, no baseline factors—including age, clinical severity (SCCAI), endoscopic severity (UCEIS), or number of prior advanced therapies—were significantly associated with the requirement for re-induction (LLR *P* = 0.54). Patients undergoing extended induction (*n* = 41) exhibited numerically higher baseline SCCAI and UCEIS scores compared to the standard induction group (not statistically significant), although the number of prior lines of therapy was comparable (2.5 vs 2.3).

Among patients with follow-up data extending beyond six months, treatment persistence beyond 6 months was 83% (58 of 70). There was no significant difference in six-month persistence between patients who did and did not receive extended IV induction (*P* = 0.5) (Figure 4C). At the time of analysis, 72 patients (72%) remained on mirikizumab.

### Disease activity and biomarker response

**Figure 1:**
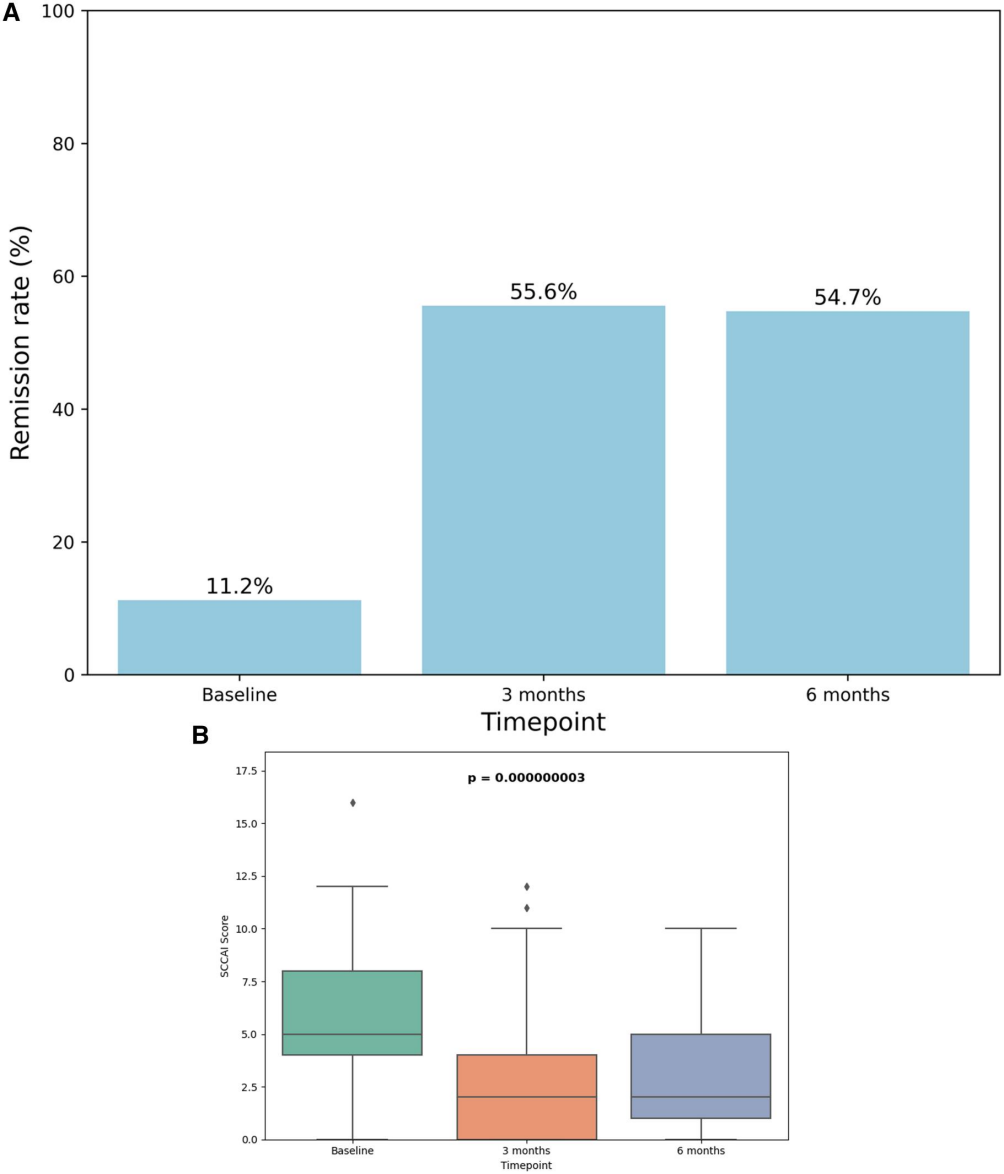
(A) Rates of clinical remission. proportion of patients achieving clinical remission (defined as SCCAI ≤ 2) at baseline, 3 months (*n* = 82), and 6 months (*n* = 52). (B) Mean SCCAI score at baseline, 3 months (*n* = 82) and 6 months (*n* = 52).

The SCCAI score decreased significantly from a baseline mean of 5.8 (SD: 3) to 3.3 (SD: 1.4) at three months and 3.7 (SD: 1.5) at six months (*P* < .00001 for both). 10/89 (11%) patients had SCCAI score ≤2 when starting mirikizumab. 50/90 (56%) achieved clinical remission at 3 months (SCCAI score ≤2), 29/53 (55%) achieved clinical remission at 6 months (Table 2). The clinical response rate increased from 53.7% at 3 months to 61.5% at 6 months (Figure 1).

**Figure 2:**
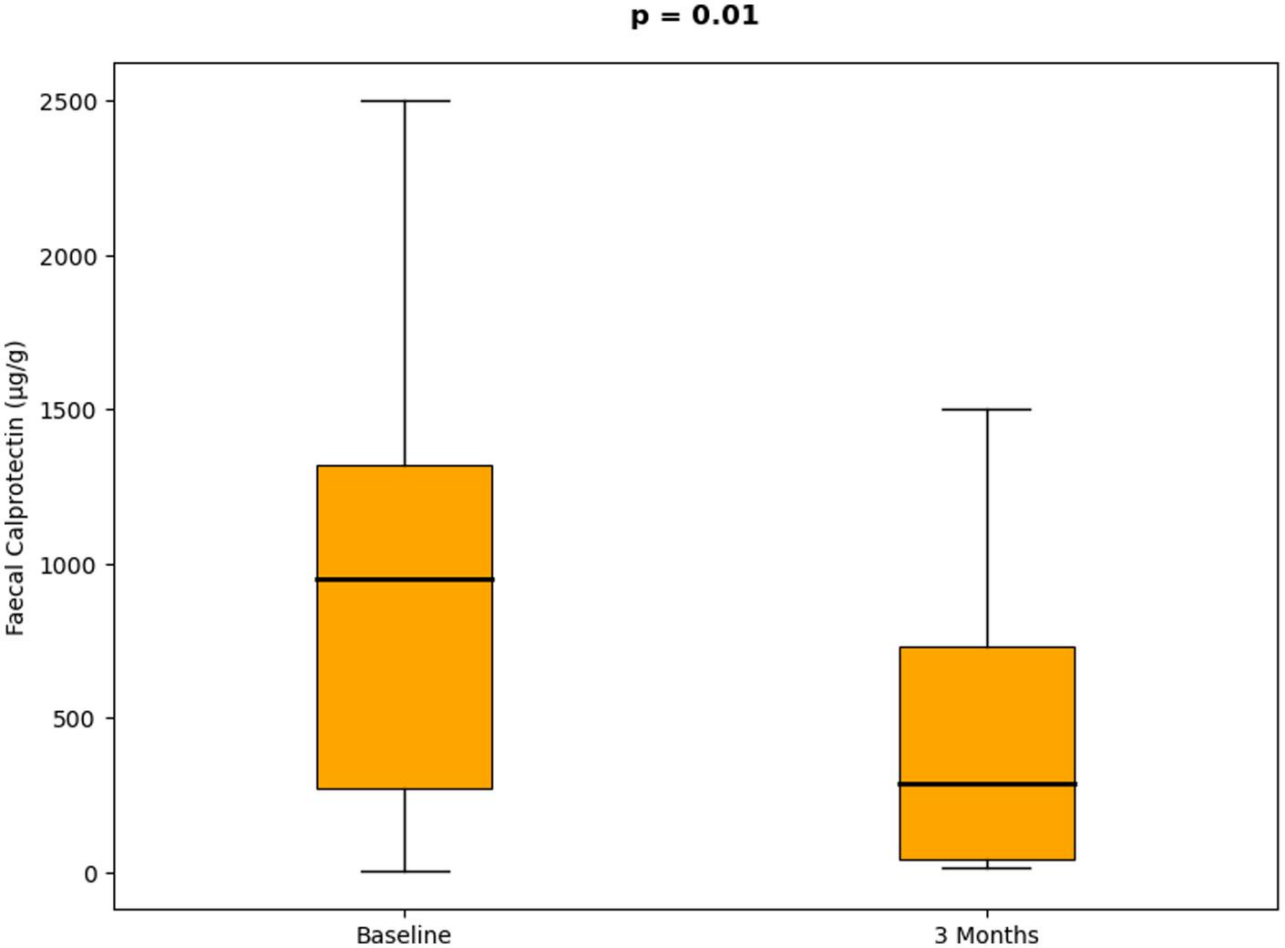
Reduction in mucosal inflammation. Box-and-whisker plots (median, IQR and range) representing the distribution of fecal calprotectin levels at baseline and 3 months (*n* = 43).

Fecal calprotectin also fell from 1094 µg/g (SD: 1122) at baseline to 586 µg/g (SD: 688, *n* = 43) at three months (*P* = 0.006) (Figure 2). CRP levels did not change significantly during follow-up.

**Figure 3:**
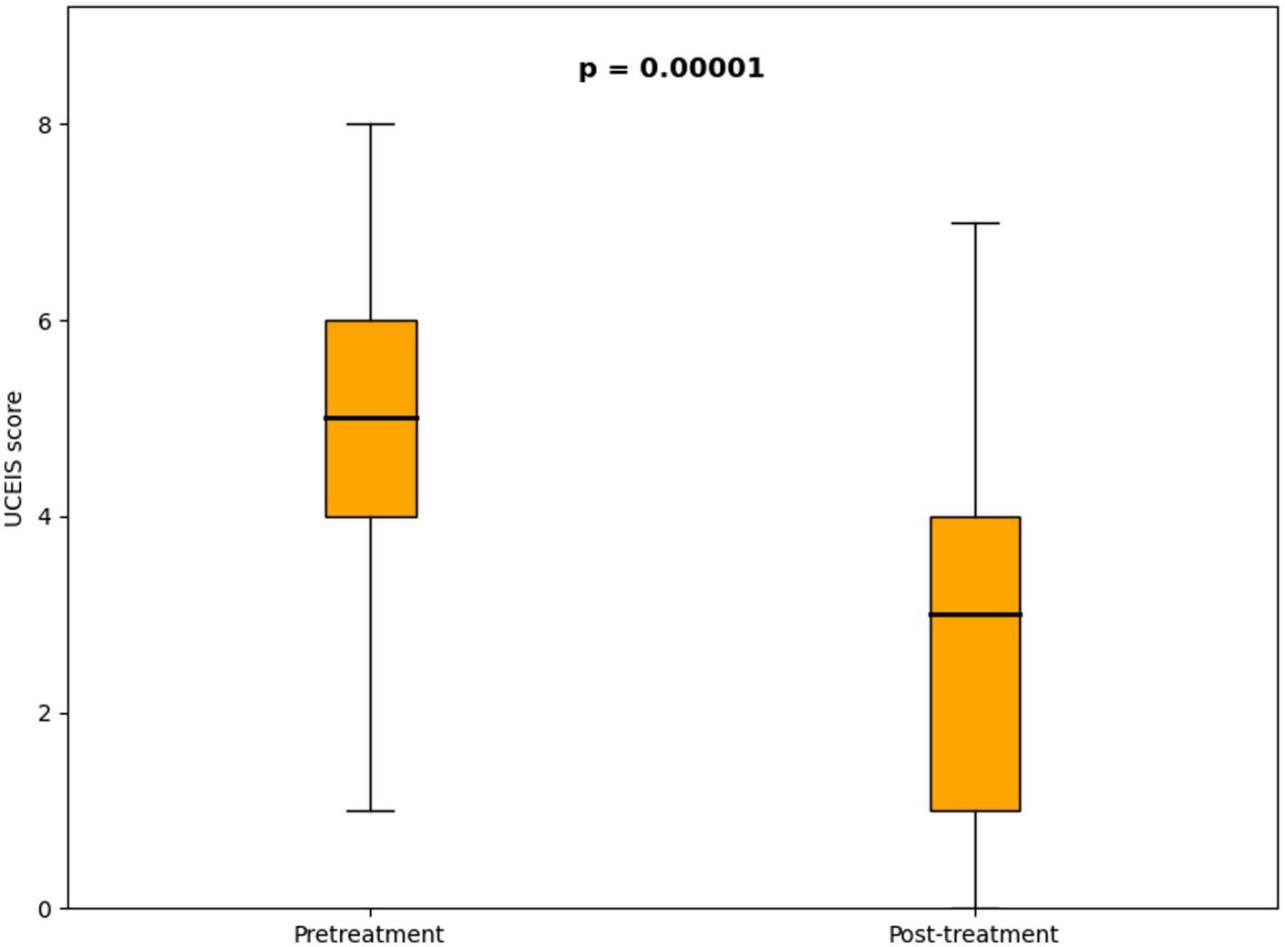
UCEIS scores pretreatment (*n* = 74) and post-induction (*n* = 52). Wilcoxon signed rank *P* = 0.00001 (*n* = 36) (box and whisker plots represent median, IQR and range).

Paired endoscopic data, available in 36 patients, showed a significant reduction in UCEIS scores from 5 (SD: 1.5) pretreatment to 3 (SD: 1.4) at least three months after therapy initiation (*P* = .000001) (Figure 3). Post-treatment endoscopy was conducted between 3- and 7-month following initiation of mirikizumab therapy.

Comparative analysis revealed no significant differences in baseline age or prior treatment burden between patients with and without complete follow-up data. Patients with complete 6-month data had numerically higher baseline disease activity (SCCAI 6.5 vs 5.0), suggesting that attrition was not biased toward the exclusion of more severe cases.

### Influence of prior ustekinumab exposure

Among those with prior ustekinumab exposure (*n* = 36), 26 (72%) continued therapy, compared to 46/64 (72%) in patients without prior ustekinumab exposure (*P* = 1.0). The duration of persistence did not significantly differ based on prior biologic exposure nor with the number of previous therapies.

Patients previously exposed to ustekinumab (*n* = 36) had comparable improvements in clinical and endoscopic measures to those without prior exposure; there were no significant differences in baseline characteristics in terms of disease activity (UCEIS, SCCAI, CRP *P* > .05).

### Influence of prior anti-TNF exposure

**Figure 4:**
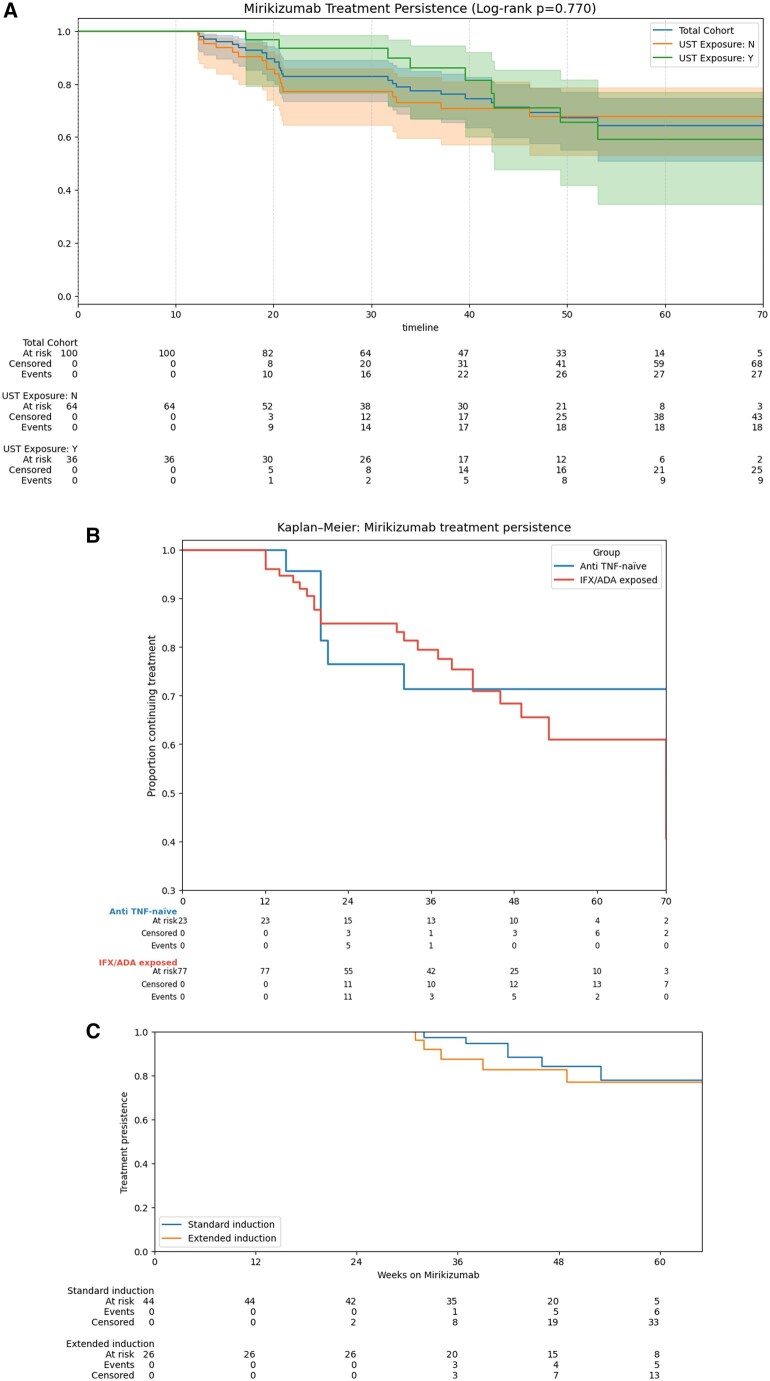
Effect of previous treatment exposure and extended induction on treatment persistence. (A) Kaplan–Meier survival curves demonstrating treatment persistence over a 70-week timeline for the total cohort (*n* = 100, blue line), ustekinumab-naïve patients (*n* = 64, orange line), and ustekinumab-exposed patients (*n* = 36, green line). (B) Persistence by anti-TNF status. Comparison of treatment survival between anti-TNF exposed (*n* = 77, orange line) and anti-TNF naïve (*n* = 23, blue line) cohorts, showing comparable durability (*P* = .30). (C) Persistence by Induction Strategy. Comparison of treatment persistence beyond 6 months comparing patients who received standard (*n* = 44, blue line) or extended (*n* = 26, yellow line) intravenous induction (*P* = .50).

In patients previously exposed to anti-TNF (*n* = 77), there was no statistical difference in treatment persistence compared with the TNF-naïve group. The median treatment survival in anti-TNF-exposed patients was 37 weeks, compared with 44 weeks in the anti-TNF-naïve group (*P* = .3) (Figure 4B).

### Influence of other advanced therapy exposure

There was no statistical difference in treatment persistence with mirikizumab in patients with previous vedolizumab (*P* = .82) or JAK-inhibitor (*P* = .79) exposure. In addition, there was no statistical difference in treatment persistence rates among patients who were exposed to 3 or more biologics compared with those who were exposed to fewer than 3 biologics (*P* = .22), regardless of ustekinumab exposure (data not shown).

**Figure 5:**
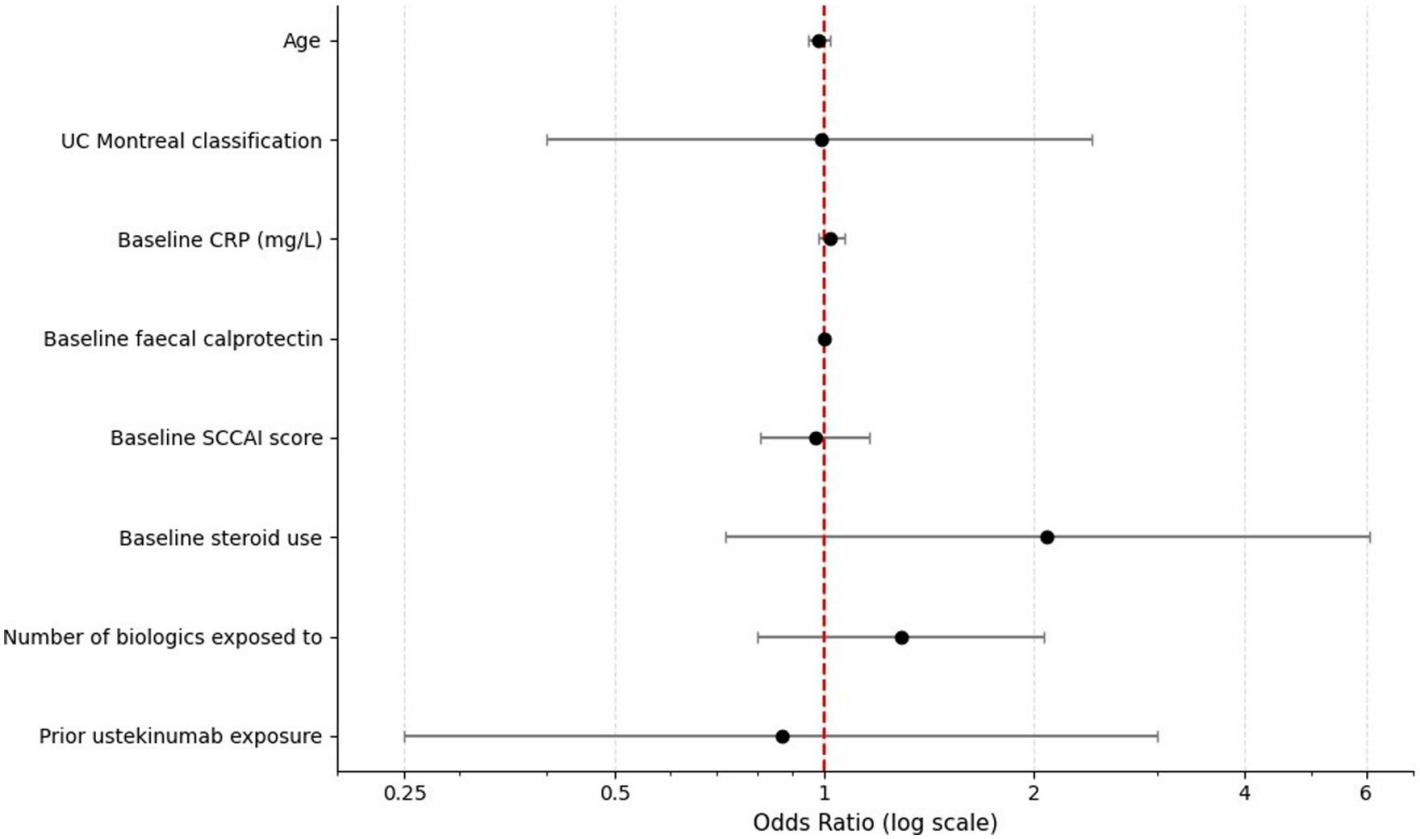
Multivariable logistic regression analysis of baseline factors associated with 12-month mirikizumab persistence.

Multivariable logistic regression did not identify baseline factors significantly associated with treatment persistence (Figure 5). However, given the relatively low number of discontinuation events, the wide confidence intervals suggest the model may be underpowered to detect subtle clinical predictors.

### Safety and adverse events

Only 6 patients reported mild adverse effects including muscle aches (*n* = 1), skin rashes (*n* = 2), transaminitis (*n* = 1), and unspecified effects (*n* = 2). None of these events led to treatment discontinuation, and no serious infections, infusion reactions, or cases of true liver dysfunction were reported. Side effects did not differ between ustekinumab-exposed and ustekinumab-naïve patients.

Colectomy was performed in 8 patients (8.0%) at a median of 20.6 weeks after initiation. These patients represented a highly refractory subgroup with a mean of 4.0 prior advanced therapy failures (compared to 2.4 in the non-colectomy group) and severe baseline endoscopic activity (mean UCEIS 6.4). No patients in the surgery subgroup demonstrated initial clinical or biochemical response to mirikizumab induction.

## Discussion

This retrospective multicenter cohort study describes the largest real-world cohort to date, with the longest follow-up, highlighting the effectiveness and safety of mirikizumab in the management of UC. In addition, we present the first assessments of mucosal response measured indirectly with fecal calprotectin, and directly with endoscopy.

Our findings in the ustekinumab-naïve cohort largely reflect those seen in the phase 3 LUCENT trials of mirikizumab in patients with moderate to severe UC and demonstrate that mirikizumab achieves clinical, biochemical, and endoscopic improvements in a treatment-refractory, real-world population. Importantly, more than a third of patients in this study had been exposed to ustekinumab prior to mirikizumab and were found to have similar responses to the ustekinumab-naïve cohort.

A smaller Japanese cohort of patients (*n* = 52) has recently been published, but the applicability of these data is limited by the fact that many of the patients were also being treated with tacrolimus and no endoscopic data were presented.^11^ In addition, like two very small cohorts from centers in the US (*n* = 22) and Japan (*n* = 10), follow-up was limited to 12 weeks.^12^^,^^13^ None of the studies looked at the impact of extended induction on the treatment outcome, whereas our findings showed extended induction does not significantly influence treatment survival beyond six months.

The majority of our patients were exposed to anti-TNF drugs, only 18 patients being anti-TNF naïve. There was no statistical difference in treatment survival among patients exposed to anti-TNF compared with the TNF-naïve group. The LUCENT trials have demonstrated that mirikizumab is an effective therapy for UC, including in patients who have previously been treated with an anti-TNF.^1^ Sub-group analysis demonstrated that in patients with one anti-TNF failure, significantly more patients achieved clinical response at week 12 compared to placebo (64.4% vs 34.1% *P *= .001), clinical remission at week 52 (44.3% vs 17.2%, *P *= .017), and symptomatic remission at week 52 (63.9% vs 34.5%, *P *= .005).^14^

Treatment persistence is an important surrogate for both drug effectiveness and tolerability in the real-world setting. Our study demonstrates high real-world mirikizumab durability, with 64% persistence at 1 year. By utilizing time-to-event and landmark analyses, we account for discontinuations occurring after corticosteroid tapering, which typically follows the initial 3-month induction. The nearly identical persistence curves between ustekinumab-exposed and naïve groups (*P* = .77) suggest that IL-23p19 inhibition remains effective despite prior IL-12/23 failure. The effectiveness rates are comparable to those reported in the mirikizumab registration trials, but in a more treatment refractory cohort, 45% of the patients in our study had been exposed to 3 or more AT and would have been excluded from the LUCENT studies.^1–3^

While treatment persistence is a robust real-world endpoint, we acknowledge that in highly refractory populations, it may occasionally reflect a lack of therapeutic alternatives or delayed surgical decision-making. However, the concurrent and significant improvements observed in both SCCAI scores and fecal calprotectin levels suggest that persistence in this cohort was underpinned by objective clinical and biochemical response.

The rapid reduction in both SCCAI and fecal calprotectin within 12 weeks (*P* < .001 and *P* = .006, respectively) suggests that mirikizumab provides a swift therapeutic onset, even in a multi-refractory cohort. Moreover, endoscopic scores, where measured, decreased significantly, again reflecting the mucosal improvements seen in the clinical trial programme.^10^^,^^15^ The reduction in fecal calprotectin and mucosal inflammation was not reflected by a reduction in CRP, which likely reflects the limitations of CRP as a biomarker in UC.^16^^,^^17^

The question of whether previous treatment with ustekinumab influences subsequent response to mirikizumab is highly relevant to clinical practice, particularly since the introduction of biosimilar ustekinumab and its subsequent use earlier in the treatment algorithm in UC. Patients previously exposed to ustekinumab were excluded from the LUCENT trials as well as from the phase 3 programs for risankizumab and guselkumab in UC. Our data identified no significant difference in treatment persistence or clinical outcomes in patients with and without prior ustekinumab exposure; the cohorts were similar in terms of baseline characteristics apart from generally greater AT exposure in the ustekinumab-experienced patients. Interestingly, albeit in our relatively small cohort of 36 patients, response rates were not diminished in ustekinumab-exposed patients who had also received two or more other AT compared with those who had been exposed to only one other AT in addition to p40. This is the largest ustekinumab-exposed cohort of p-19-treated UC patients published to date and the only one with follow-up beyond 12 weeks. The results not only reflect the smaller Japanese cohort but also those seen with the use of risankizumab in ustekinumab-exposed patients with Crohn’s disease or psoriasis patients, both in a clinical trial and in a real-world setting.^7^^,^^8^^,^^18^^,^^19^

IL-23 plays a central role in Th17-driven mucosal inflammation.^20^ The fact that patients previously treated with ustekinumab, which inhibits IL-12/23 via p40, respond to IL-23p19 blockade suggests the persistence of IL-23-driven pathogenicity independent of IL-12 pathways.^21^ This supports the concept that blockade of the IL-23 p19 subunit may be more important to inhibit Th17-mediated inflammation, where IL-12 may have a protective role in inflammation.^22^

Mirikizumab was well tolerated overall, with adverse events being infrequent and mild. No serious infections or adverse safety signals emerged, aligning with the safety profile reported in clinical trial data.^1–3^ The low incidence of side effects provides further support for the safety of IL-23 inhibition in patients with IBD.^23^

Our study has several strengths, including its multicenter design as well as access to prospectively collected clinical data. As with most such cohorts, there are, however, several limitations. To maintain patient anonymity and comply with the data governance standards of a clinical service evaluation, certain demographic, and historical parameters (e.g., gender, BMI, and specific extraintestinal manifestations) were not extracted. The lack of these variables is a recognized limitation of this real-world dataset. The retrospective nature of the study introduces inherent biases although these are, in part, mitigated by the fact that all data were collected prospectively, and all patients treated with mirikizumab with appropriate follow-up were included. Variability in assessment timing and measures, as well as incomplete coverage of some outcome measures, were also notable limitations. However, the similarity in baseline characteristics and prior therapy exposure between complete and incomplete cases suggests that significant attrition bias is unlikely. The relatively short follow-up period (median of 37 weeks) limits understanding of long-term response durability, relapse rates, and safety profile but is significantly longer than any other cohort described to date. Additionally, the cohort predominantly included patients with moderate to severe disease refractory to conventional therapies treated in tertiary referral centers, thus reflecting only a subset of patients with UC. Another potential limitation is that mirikizumab has a license for extended induction, whereby non-responders at week 12 can receive a further 12 weeks of treatment. The response-driven induction strategies, which may introduce practice-level heterogeneity compared to the rigid 12-week induction used in clinical trials. This might mean that for some patients, treatment persistence at 3 months was not reflective of ultimate response to therapy. Nevertheless, we did not detect a significant difference in treatment persistence at 6 months between patient groups based upon receipt of extended IV induction.

Finally, the need for intensified dosing (extended induction or re-induction) was not strictly confined to the most treatment-refractory patients (those failing the highest number of biologics) and could not be predicted using baseline variables.

Accordingly, the real-world data presented here complement existing trial data and suggest that IL-23p19 inhibition with mirikizumab remains efficacious both before and after IL-12/23 pathway blockade. Furthermore, like in the clinical trial setting, symptomatic responses are reflected by improvement in mucosal inflammation, which is known to be associated with improved long-term outcomes. Further larger studies with longer-term follow-up will further improve our understanding of the role IL-23 inhibition in UC.

## Data Availability

The datasets generated and/or analyzed during the current study are not publicly available due to the use of identifiable real-world clinical data obtained under institutional approvals that do not permit public deposition. Requests for access to de-identified data may be considered by the study investigators and sponsoring institution, subject to governance approvals and applicable data protection regulations.
